# FAP-targeted [^68^Ga]BED003-PET in different solid malignancies

**DOI:** 10.1007/s00259-026-07842-1

**Published:** 2026-03-19

**Authors:** David Ventura, Michael Schäfers, Stefanie Bobe, Florian Büther, Jonas Brandt, Lars Stegger, Philippe Brüggemann, Wolfgang Roll, Kambiz Rahbar, Philipp Schindler, Philipp Backhaus

**Affiliations:** 1 Department of Nuclear Medicine, University Hospital Münster, Münster, Germany; 2 European Institute for Molecular Imaging (EIMI), University of Münster, Münster, Germany; 3 West German Cancer Center (WTZ), Münster Site, Münster, Germany; 4 Gerhard Domagk Institute of Pathology, University Hospital Münster, Münster, Germany; 5 Clinic of Radiology, University and University Hospital Münster, Münster, Germany

**Keywords:** OncoFAP, FAPI, PET, Solid Malignancies, Radioligand therapy

## Abstract

**Purpose:**

Positron emission tomography (PET) using fibroblast activation protein inhibitors (FAPI) has emerged as a robust imaging tool for solid tumours. The novel ligand [^68^Ga]BED003 (formerly known as [^68^Ga]Ga-OncoFAP-DOTAGA) has demonstrated very high affinity for the fibroblast activation protein and favourable biodistribution. This study aimed to assess the in vivo distribution of [^68^Ga]BED003 across a spectrum of solid tumours as a potential prerequisite for radioligand therapy.

**Methods:**

In this retrospective analysis, [^68^Ga]BED003 PET/CT or PET/MRI of 157 patients with 19 different solid malignancies were retrospectively analysed. A spherical volume of interest (VOI) was placed over the primary tumour, the most intense lymph node and distant metastases that were evaluated as at least likely malignant in the written reports, and the maximum standardized uptake value (SUV_max_) was derived. Liver and blood pool background mean SUV (SUV_mean_) were assessed using standardised VOIs. Tumour-to-background ratios (TBR_max_) were calculated as the ratio of SUV_max_ to background SUV_mean_. SUV_max_ and TBR_max_ values of primary tumours, lymph node, and distant metastases were compared using Wilcoxon rank-sum and signed-rank tests, as appropriate.

**Results:**

Overall, 115 primary tumours, 70 lymph node metastases, and 116 distant metastases were analysed, yielding median SUV_max_ of 16.6, 12.3, and 11.9, respectively. No significant difference in SUV_max_ or TBR_max_ were observed between lymph node and distant metastases (all *P* > 0.05). In contrast, primary tumours demonstrated significantly higher uptake than lymph node and distant metastases (all *P* < 0.001), except for liver TBR_max_ when comparing primary tumours with lymph node metastases (*P* > 0.05). Across all 301 lesions, the median SUV_max_, liver TBR_max_ and blood pool TBR_max_ was 14.7 (range, 4.2–35.9), 21.6 (range, 4.2–62.8) and 11.1 (range, 3.0–33.4), respectively. The highest median SUV_max_ and TBR_max_ (both) in cohorts with > 5 lesions were found in medullary thyroid, oesophageal, ovarian, cervical, breast, colorectal, and hepatocellular cancers.

**Conclusion:**

[^68^Ga]BED003-PET demonstrated consistently high uptake across diverse solid malignancies, supporting its potential role as a tool for multi-cancer diagnostic imaging and patient selection for FAP-targeted radioligand therapy.

**Supplementary Information:**

The online version contains supplementary material available at 10.1007/s00259-026-07842-1.

## Introduction

Positron-emission tomography (PET) with radiolabelled fibroblast-activation protein (FAP) inhibitors (FAPI) has emerged as a reliable diagnostic tool for a growing number of non-oncological and oncological diseases in recent years [1]. For various solid malignancies, the presence of FAP expression has been observed in cancer-associated fibroblasts (CAFs), which are located in the tumour microenvironment [2]. In contrast, the presence of FAP expression in healthy tissue is negligible [3]. Consequently, radiolabelled FAPI ligands are promising radiotracers for molecular imaging, given their high affinity for a variety of solid malignancies and their rapid clearance in healthy organs [4]. A variety of positron-emitting (i.e. [^68^ Ga]gallium or [^18^F]fluorine) radiolabelled FAPI ligands (e.g. FAPI-04, FAPI-46, FAPI-74 or FAPI-2286) for PET were introduced [1, 5, 6]. A series of investigations have documented the remarkable tumoral tracer affinity of PET scans using radiolabelled FAPI ligands for various solid malignancies [6–10]. In comparison to the widely employed [^18^F]fluorodeoxyglucose (FDG)-PET, imaging with FAPI-PET demonstrated superior diagnostic accuracy in several solid malignancies [11].

The recently introduced new FAPI ligand [^68^Ga]BED003 (formerly known as [^68^Ga]OncoFAP-DOTAGA) (Blue Earth Diagnostics Ltd) has been shown to display superior FAP affinity compared with other FAPI tracers, as well as superior uptake in murine FAP expressing tumour models [12]. First clinical results for PET with [^68^Ga]BED003 have demonstrated a high tumoral tracer affinity with excellent tumour-to-background ratios (TBRs) in human subjects [12–15]. The prospective multicentre phase I clinical study of [⁶⁸Ga]BED003 (FAPrimo, NCT05784597), designed to evaluate safety and dosimetry, demonstrated a favourable safety profile and optimal pharmacokinetics, characterized by rapid blood clearance and predominant renal excretion [16]. Further development of radiolabelled BED003 multimers has been shown to result in a higher tracer affinity and prolonged tumour retention in tumour bearing mice [17, 18]. These radiochemical developments, involving multivalent tracers, have provided a rationale for the application of radioligand therapy approaches [19]. A clinical trial with the BED003 derivative [^177^Lu]LuOncoFAP-23 is currently ongoing (Theratri, NCT06640413).

This retrospective study aimed to extend the first clinical results of [^68^Ga]BED003-PET in a large cohort of patients with a broad spectrum of different malignancies and to explore its potential relevance for selecting patients for FAP-targeted radioligand therapy.

### Methods

#### Patients

All patients were referred for [^68^Ga]BED003-PET, following interdisciplinary tumour conference decisions in accordance with current literature evidence and/or on an individual patient basis. PET scans were performed between 03/21 and 10/25. The following inclusion criteria were applied: a) histopathological confirmed malignancy with no evidence of second malignancy; b) suspected elevated tracer uptake in reported lesions; c) exclusion of known recently locally treated lesions (e.g., radiation therapy); d) exclusion of lesions with any remission status after or under treatment; e) age > 18 years. No additional exclusion criteria were defined. Informed consent was obtained from all patients for imaging with [^68^Ga]BED003. Patients with 19 different kinds of malignancies were included. A small number of patients were already reported previously: 10 of 17 included patients with oesophageal cancer who underwent subsequent radiation treatment after the PET scan [15], 12 patients with various malignancies [12] and a case report of one patient with squamous skin cancer [14]. This retrospective study was not registered as a clinical trial and was approved by the local ethics committee of Westphalia-Lippe and the medical faculty of the University of Münster (approval no. 2025–388-f-S).

#### Radiosynthesis of [^68^Ga]BED003 ([^68^Ga]OncoFAP-DOTAGA)

**Fig. 1:**
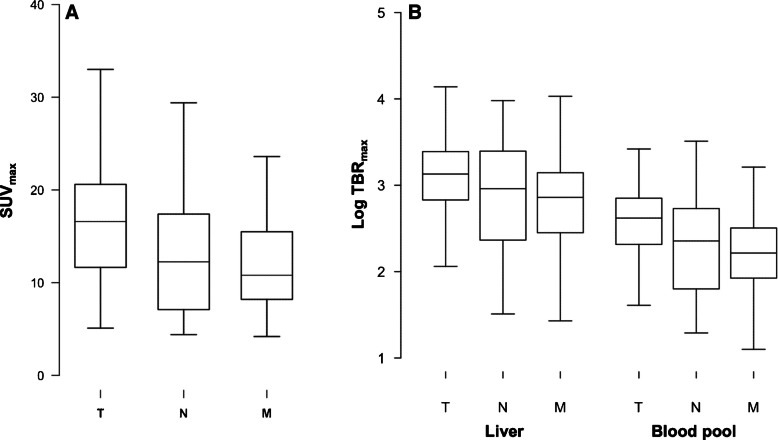
Statistical analysis demonstrated a significantly higher median SUV_max_ for primary tumours, while median SUV_max_ values were comparable between lymph node and distant metastases (A). Analysis of TBR_max_ values yielded similarly significant results, except for the comparison of liver TBR_max_ between primary tumours and lymph node metastases (B). TBRmax values were log-transformed to improve visualisation

[^68^Ga]BED003 is a small organic FAP ligand and was produced in-house in our radiochemistry department in accordance with the regulations of the German Pharmaceuticals Act §13(2b) as described in detail previously [12, 20]. The organic synthesis was provided as published previously (Fig.1S [a-b]) [20]. Owing the presence of a carboxylic acid functional group, the BED003 scaffold allows for straightforward conjugation with different payloads via standard amide coupling chemistry. The small molecule was enabled with a DOTAGA chelator (Fig. 1S [c]) that provides radiolabeling with [^68^Ga]gallium (Fig. 1S [d]) [12].

#### PET imaging

The image acquisition was performed on either a 3-Tesla PET/magnetic resonance imaging (MRI) scanner (3T Biograph mMR, Siemens Healthineers) or a PET/computed tomography (CT) scanner (Biograph 128 mCT, Siemens Healthineers). The selection of the scanner, and PET field of view were made on an individual patient basis. If performed, Gadovist® (Bayer, Germany) and Ultravist® (Bayer, Germany) were employed as contrast agents for MRI and CT, respectively. In the context of whole-body imaging with PET/MRI, the following sequences were acquired: axial T1- and T2-weighted, coronal fat-suppressed T1-weighted and optional contrast-enhanced fat-suppressed axial and coronal T1-weighted. The addition of sequences such as liver diffusion-weighted and dynamic imaging or multiparametric MRI of the breast was made based on individual clinical settings. For whole-body imaging with PET/CT, a low-dose CT scan was performed for the purpose of attenuation correction and anatomical orientation. Additional contrast-enhanced CT sequences were performed on an individual per patient basis. Images were acquired 1 h after intravenous injection of 2–2.5 MBq per kilogram body weight of [^68^Ga]BED003 with a median activity of 162 MBq (range: 129–276 MBq). Whole-body PET acquisition was performed from the skull base or skull to mid-thigh, with optional extension to the feet based on individual clinical decisions, using an acquisition time of 3 min per bed position (PET/MRI) or a continuous-bed-motion speed of 1.1 mm/s (PET/CT). PET acquisition on the PET/CT scanner was performed in accordance with the EANM Research Ltd. (EARL) procedure guidelines version 2.0 (EARL-2), whereas PET acquisition on the PET/MRI scanner was conducted in close adherence to the EARL-2 recommendations [21].

#### Image analysis

All acquired imaging data were analysed in consensus by one senior nuclear medicine and one senior radiologist physician both with > 10 years of imaging experience. In instances where established tracer cut-offs were not available, any suspect uptake above the background level was subjected to scrutiny and subsequently evaluated in comparison with morphological, prior or follow-up imaging. In this context, lesions that were possible to classify as benign (e.g., liver haemangioma) were excluded. All lesions included into this analysis were classified malignant. The maximum standardized uptake value (SUV_max_) was obtained from lesions classified as malignant with a 40% SUV-body weight cut-off volume of interest (VOI) using the imaging software Syngo.Via (Siemens Healthineers). The SUV_max_ was collected for the following: known primary tumour; one lymph node metastasis of any location, and one lesion per organ system (distant metastases) with the highest tracer uptake each, if applicable. For background measurements, an isovolumetric spherical VOI of 3.5 mL was placed in unaffected liver tissue (if applicable), and a cylindrical VOI of 1.5 mL was positioned within the thoracic aorta to assess blood pool activity. Maximum TBR (TBR_max_) were calculated as the ratio of lesional SUV_max_ to the corresponding background mean SUV (SUV_mean_) for liver and blood pool, respectively.

#### Statistical analysis

All statistical analyses were performed using RStudio (R version 4.5.2). Continuous variables are reported as median, range or 95% confidence interval (95% CI). Due to non-normal data distribution, non-parametric tests were applied throughout. Patient-based comparisons of SUV_max_, liver TBR_max_, and blood pool TBR_max_ between lesion categories and between imaging modalities (PET/MRI vs PET/CT) were performed using the Wilcoxon rank-sum test. Lesion-based paired comparisons between primary tumours and the most avid metastatic lesion within the same patient were assessed using paired Wilcoxon signed-rank tests, with effect sizes calculated as *r*. To account for multiple lesions per patient, lesion-based linear mixed-effects models with log-transformed SUV_max_, liver TBR_max_, or blood pool TBR_max_ as dependent variables were fitted using the lme4 package, including imaging modality as a fixed effect and patient identification number as a random intercept; statistical inference was obtained using Satterthwaite’s approximation implemented in the lmerTest package, and model estimates were back-transformed to relative percentage differences. Data handling was performed using dplyr and tidyr packages, and figures were generated with ggplot2 package. A two-sided *P *value < 0.05 was considered statistically significant.

## Results

### Patient-based analysis

A total of 157 patients (88 females and 69 males; median age 61 years) were available for analysis. No relevant adverse events after tracer injection were reported. Overall, 54 patients (34.4%) were examined using PET/MRI, with 50/54 (92.6%) receiving a contrast agent, while 103 patients (65.6%) underwent PET/CT, of whom 48/103 (46.6%) received a contrast agent. A total of 115 primary tumours, 70 lymph node metastases and 116 distant metastases were investigated. The median liver SUV_mean_ was 1.23 (range, 0.67–1.94) compared with a median blood pool SUV_mean_ of 0.66 (range, 0.33–2.01). The corresponding median SUV_max_, liver TBR_max_ and blood pool TBR_max_ values are summarized in Table 1.

**Table 1:** Overview of median SUV_max_, median liver TBR_max_ and blood pool TBR_max_ values

	Median SUV_max_	95%CI	Median liver TBR_max_*	95%CI	Median blood pool TBR_max_	95% CI
Primary tumours (*n* = 115)	16.6	15.9–18.3	22.8	21.2–24.35	13.7	12.3–14.7
Lymph node metastases (*n* = 70)	12.3	11.7–14.9	19.4	14.2–23.3	10.5	8.5–11.7
Distant metastases (*n* = 116)	11.9	11.1–12.9	17.4	15.2–19.4	9.2	8.5–10.2

^*^5 patients were excluded due to chronic liver disease

The SUV_max_, liver TBR_max_ and blood pool TBR_max_ did not differ significantly between lymph node and distant metastases, (*P* = 0.64, *P* = 0.21 and *P* = 0.29), respectively. In contrast, the SUV_max_, liver TBR_max_, and blood bool TBR_max_ of primary tumours were significantly higher than those of lymph node metastases and distant metastases (all *P* < 0.001), except for the comparison of liver TBR_max_ between primary tumours and lymph node metastases, which did not reach statistical significance (*P* = 0.059). The highest SUV_max_, liver and blood pool TBR_max_ per patient did not differ significantly between PET/MRI and PET/CT (*P* = 0.73, *P* = 0.75 and *P* = 0.74). The boxplot for SUV_max_, log-transformed liver TBR_max_ and blood pool TBR_max_ of all primary tumours (T), lymph nodes (N) and distant metastases (M) is shown in Fig. 1.

**Fig. 2:**
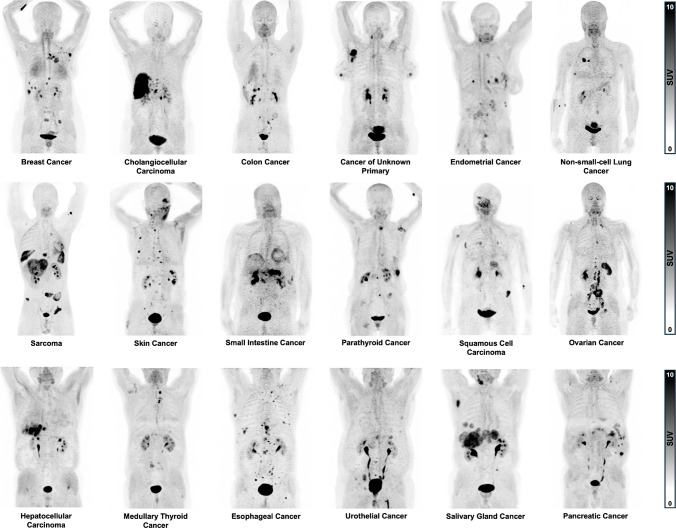
Visual comparison of maximum intensity projection images from [^68^Ga]BED003-PET in 18 patients with different solid malignancies

The highest median SUV_max_ in malignancies with more than five analysed lesions available was observed in cases of medullary thyroid, oesophageal, hepatocellular, ovarian, cervical and breast cancer. The lowest median SUV_max_ was observed in endometrial cancer and squamous cell carcinoma of the skin. Figure 2 provides an overview of [^68^Ga]BED003 distribution across a range of entities by maximum intensity projections.

### Lesion-based analysis

All investigated entities demonstrated a sufficient lesional median SUV_max_ of 14.7 (*n* = 301; range, 4.2–35.9). Comparison on a lesion-based level for each patient demonstrated significantly higher SUV_max_ for the primary tumour compared with the SUV_max_ of the most avid lymph node metastasis (*n* = 48; *P* < 0.001; *r* = 0.55), lung metastasis (*n* = 10; *P* = 0.019; *r* = 0.74), liver metastasis (*n* = 15; *P* = 0.009; *r* = 0.66), and other distant metastases (e.g. brain, peritoneum; *n* = 16; *P* = 0.038; *r* = 0.51), all exhibiting large effect sizes (*r* > 0.5). In contrast, the lesion-based comparison between the primary tumour and the most avid bone metastasis did not reach statistical significance (*n* = 13; *P* = 0.345), showing a small effect size (*r* = 0.26). Comparison of imaging modality (PET/MRI vs PET/CT: 91 vs 210 lesions) demonstrated no significant difference for SUV_max_ between both scanners (estimate −0.036 on the log scale [-3.6% difference in favor for PET/MRI]; *P* = 0.58). A comprehensive overview for individual entities and entity-based SUV_max_ measurements is presented in Table 2 and more detailed in Table 1S.

**Table 2:** Overview for SUV_max_ values of separated entities

Entity	n (%)	Lesions	Median SUV_max_	Range SUV_max_
Breast Cancer	30 (19.1)	56	15.5	4.8–33.2
Pancreatic Cancer	28 (17.8)	54	13.4	4.5–29.7
Esophageal Cancer	17 (10.8)	25	16.7	4.4–28.8
Hepatocellular Carcinoma	15 (9.6)	24	13.6	5.0–28.2
Cholangiocellular Carcinoma	11 (7.0)	25	15.6	6.8–35.9
Ovarian Cancer	10 (6.4)	17	15.3	6.5–22.9
Colorectal Cancer	9 (5.7)	20	13.3	5.2–23.6
Medullary Thyroid Cancer	6 (3.8)	12	19.7	5.6–27.5
Sarcoma	6 (3.8)	14	12.5	5.2–21.6
Salivary Gland Cancer	5 (3.2)	12	14.6	8.9–22.6
Cervical Cancer	5 (3.2)	9	17.8	5.0–31.4
Skin Cancer	4 (2.5)	11	11.1	5.1–20.4
Endometrial Cancer	3 (1.9)	7	10.8	6.7–14.6
Squamous Cell Carcinoma of the skin	2 (1.3)	6	10.5	4.2–15.7
Cancer Of Unknown Primary	2 (1.3)	2	17.6	15.6–19.5
Non-Small-Cell Lung Cancer	1 (0.6)	2	19.7	18.1–21.3
Parathyroid Cancer	1 (0.6)	3	12.9	9.4–19.5
Small Intestine Cancer	1 (0.6)	1	18.3	-/-
Urothelial Cancer	1 (0.6)	1	10.4	-/-
**Total**	**157**	**301**	**14.7**	**4.2–35.9**

All investigated entities demonstrated high TBR_max_ values, with median liver TBR_max_ significantly exceeding blood pool TBR_max_ (21.6 [4.2–62.8] vs 11.1 [3.0–33.4], *P* < 0.001). Lesion-based TBR_max_ analysis revealed similar uptake patterns to those observed for SUV_max_. Primary tumours demonstrated significantly higher TBR_max_ values than lymph node and distant metastases for both liver and blood pool background measurements (e.g., blood pool TBR_max_ for primary tumour vs lymph node metastasis, *n* = 48, *P* < 0.001; *r* = 0.55). As in the SUV_max_ analysis, comparisons involving bone metastases did not reach statistical significance, indicating comparable relative behaviour across quantitative metrics (e.g., blood pool TBR_max_ comparison, *n* = 13, *P* = 0.15; *r* = 0.29). Detailed paired analysis is demonstrated in Table 2S. Liver TBR_max_ values did not differ between PET/CT and PET/MRI (estimate −0.008 on the log scale [-0.8% difference in favour of PET/MRI]; *P* = 0.798). Similarly, blood pool TBR_max_ values showed no significant scanner-related differences (estimate −0.024 on the log scale [-2.4% difference in favour of PET/MRI]; *P* = 0.435). A comprehensive overview for individual entities and entity-based TBR_max_ measurements is presented in Table 3 and more detailed in Table 3S.

**Table 3:** Overview of TBR_max_ values for separated entities

Entity (number of lesions)	Median liver TBR_max_*	Range*	Median Blood pool TBR_max_	Range
Breast Cancer (56)	24.7	7.9–55.4	11.1	3.7–33.4
Pancreatic Cancer (54)	14.6	5.4–40.1	10.1	3–24.6
Oesophageal Cancer (25)	22.7	5.2–58.8	14.2	3.6–24.2
Hepatocellular Carcinoma (24)	16.4	4.2–32.2	11.5	3.9–21
Cholangiocellular Carcinoma (25)	17.5	4.6–62.8	12.4	4.1–20.8
Ovarian Cancer (17)	23.9	11–45.8	10.1	5.6–16
Colorectal Cancer (20)	20.8	6.7–56.2	11.5	4.3–31.1
Medullary Thyroid Cancer (12)	16.1	3.6–35.7	8.8	5.8–22.2
Sarcoma (14)	19.2	5.4–30	10.6	3.8–17.5
Salivary Gland Cancer (12)	24.9	17.5–44.3	12.4	8.2–24.8
Cervical Cancer (9)	24.6	7.6–54.1	10.5	4.5–19.7
Skin Cancer (11)	14.7	6.7–29.8	8.4	3.8–17.5
Endometrial Cancer (7)	17.2	8.8–34.4	8.6	4.4–17
Squamous Cell Carcinoma of the skin (6)	18.4	7.1–33.3	10.5	4.4–17.2
Cancer Of Unknown Primary (2)	31	27.9–34.2	15.1	10.8–19.3
Non-Small-Cell Lung Cancer (2)	43.8	40.2–47.3	15.6	14.4–16.9
Parathyroid Cancer (3)	22.6	21.9–45.4	7.8	7.5–15.6
Small Intestine Cancer (1)	20.5	-/-	14.6	-/-
Urothelial Cancer (1)	16.5	-/-	7.4	-/-
**Total (301)**	**21.6**	**4.2–62.8**	**11.1**	**3.0–33.4**

^*^5 patients were excluded due to chronic liver disease

### Non-tumour-related and physiological uptake patterns of [^68^Ga]BED003

**Fig. 3:**
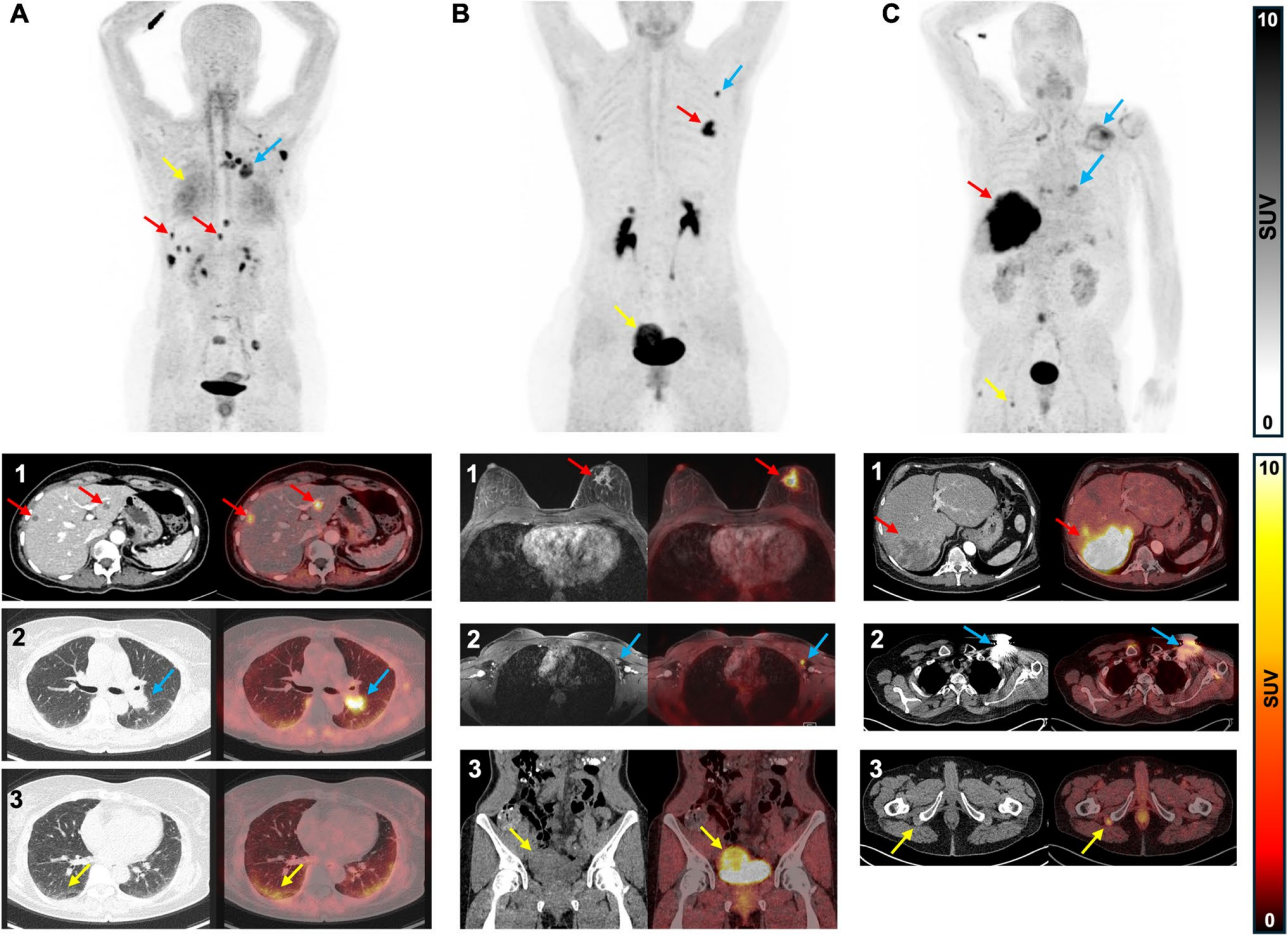
A 48-year-old female patient with metastatic breast cancer (**A**). Arterial-phase contrast-enhanced abdominal CT demonstrated hepatic (A1) and non-contrast enhanced CT demonstrated pulmonary metastases (A2) with intense fibroblast activation (red arrows, SUV_max_ up to 12.7; blue arrows, SUV_max_ up to 10.7). Increased fibroblast activation was also observed in fibrotic lung tissue (A3, yellow arrows, SUV_max_ up to 5.4). A 45-year-old female patient with breast cancer and lymph node metastasis (**B**). T1-weighted fat-suppressed MRI revealed a primary tumour in the right breast (B1) and a solitary lymph node metastasis in the left axillary region (B2), both showing intense contrast enhancement and fibroblast activation (red arrows, SUV_max_ 13.6; blue arrows, SUV_max_ 9.7). In premenopausal women, intense fibroblast activation of the uterus represents a physiological finding, as demonstrated on coronal non-contrast-enhanced CT (B3, yellow arrows, SUV_max_ up to 9.8). A 72-year-old male patient with cholangiocellular carcinoma (**C**). Arterial-phase contrast-enhanced CT revealed a large lesion in the right hepatic lobe with marginal arterial hyper perfusion and intense fibroblast activation (C1, red arrows, SUV_max_ 31.6). An implanted cardioverter-defibrillator due to dilated cardiomyopathy showed diffuse fibroblast activation, likely related to an immunogenic foreign material reaction (C2, blue arrows, SUV_max_ 9.2), as well as increased fibroblast activation in the basal left ventricular myocardium, probably reflecting fibrosis (blue arrows, SUV_max_ up to 5.6). Additionally, focal fibroblast activation was observed in the tendon of the biceps femoris muscle (C3), likely related to mechanical stress or degenerative changes (yellow arrows, SUV_max_ 7.1)

**Fig. 4:**
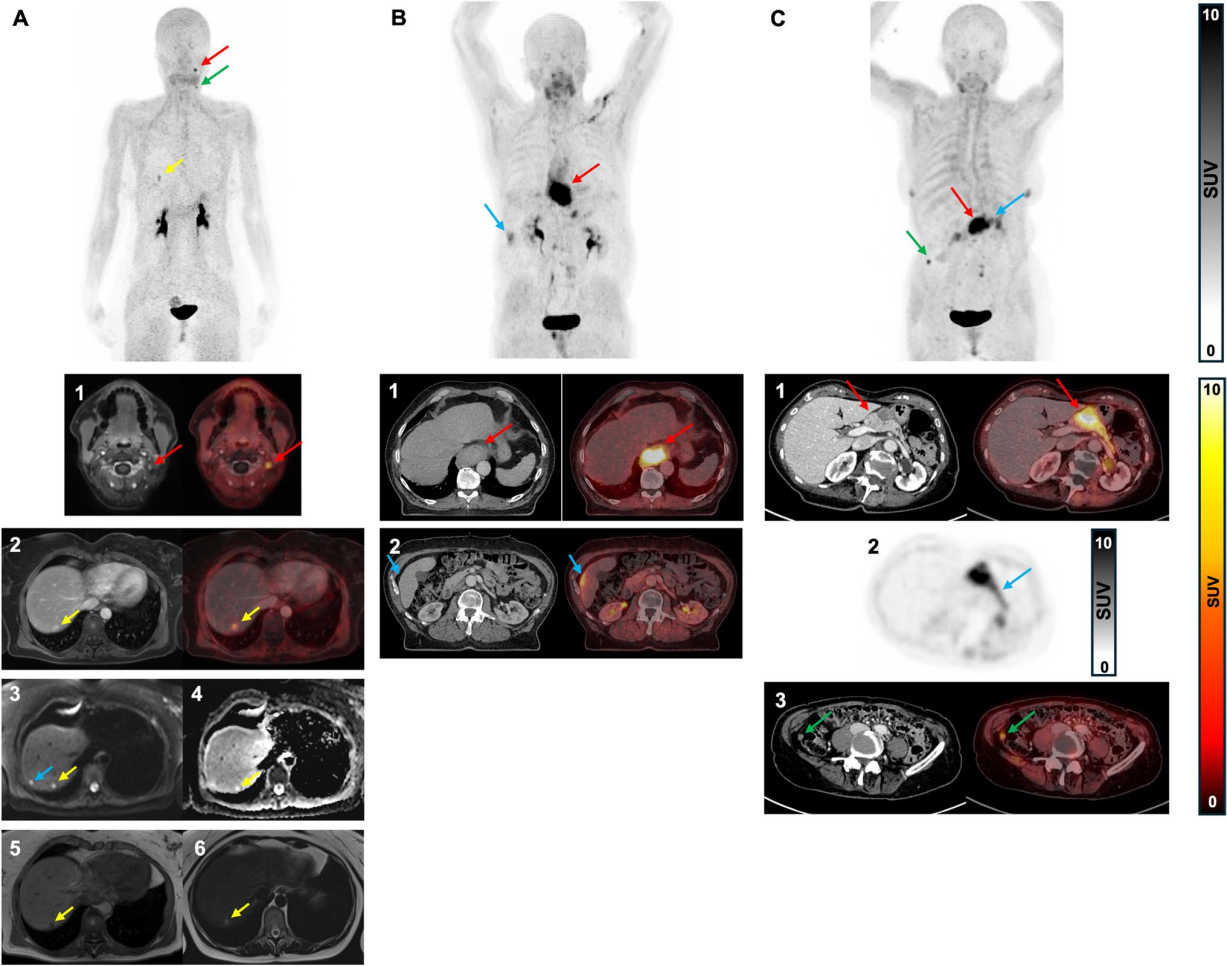
Case examples with tumoral and non-tumoral related fibroblast activation. A 52-year-old female patient with medullary thyroid carcinoma (**A**). Contrast-enhanced T1-weighted fat-suppressed MRI demonstrated a contrast-enhancing lymph node metastasis in the left cervical level IIb with fibroblast activation (A1, red arrows, SUV_max_ 9.1) and an additional lymph node metastasis in the left cervical level III (green arrow, SUV_max_ 7.2). Multiparametric MRI classified a liver haemangioma with peripheral contrast enhancement on fat-suppressed T1-weighted imaging (A2), no diffusion restriction on DWI/ADC (A3/4), and T1-weighted hypointensity (A5) and T2-weighted hyperintensity (A6), showing moderate fibroblast activation that was lower than that of the lymph node metastases (A2-A6, yellow arrows, SUV_max_ 5.5). In addition, a second liver haemangioma was identified without fibroblast activation (A3, blue arrow). A 73-year-old male patient with esophageal carcinoma (**B**). Contrast-enhanced venous-phase CT demonstrated a large tumour mass in the distal esophageus with intense patchy focal fibroblast activation (B1, red arrows, SUV_max_ 24.3). In the setting of alcohol-related liver cirrhosis, irregular hepatic parenchyma was evident on CT (B2) with diffuse background fibroblast activation (blue arrows, SUV_max_ up to 6.6). A 64-year-old female patient with pancreatic cancer (**C**). Contrast-enhanced portal-venous phase CT demonstrated a large mass in the pancreatic corpus with fibroblast activation (C1, red arrows, SUV_max_ 14.2). Likely related to concomitant chronic pancreatitis, diffuse fibroblast activation of the pancreatic tissue was observed (C2, blue arrows, SUV_max_ up to 5.4). Peritoneal carcinomatosis was evident on contrast-enhanced portal-venous phase CT (C3) with associated fibroblast activation (green arrows, SUV_max_ 7.7)

As described previously and illustrated in the maximum intensity projections of Fig. 2, [⁶⁸Ga]BED003 is renally eliminated due to its small and hydrophilic structure [16]. Within the study cohort, non–lesion-related uptake was observed in foreign bodies (e.g., implantable cardioverter-defibrillators), degenerative musculoskeletal changes (e.g., arthrosis and tendon insertions), fibrotic alterations (e.g., hepatic or pulmonary fibrosis), chronic inflammatory conditions (e.g., pancreatitis), and in the salivary glands. In contrast, lesion-related but non-malignant uptake was predominantly identified in hepatic hemangiomas and uterine myomas. Case examples illustrating tumour-related and non–tumour-related fibroblast activation are shown in Fig. 3 and Fig. 4.

## Discussion

In this investigation [^68^Ga]BED003-PET firstly demonstrates high and consistent tumour uptake across a broad spectrum of solid malignancies, confirming its suitability for imaging FAP expression in vivo. Importantly, satisfactory uptake was observed in primary tumours, lymph node metastases and distant metastases with favourable tumour-to-background contrast and without relevant adverse events, in line with previous first-in-human and translational studies of BED003-based tracers [12, 20].

On a patient-based level, primary tumours demonstrated significantly higher SUV_max_ and TBR_max_ values than lymph node and distant metastases, with the exception of liver TBR_max_ in the comparison with lymph node metastases. No significant differences were observed between lymph node and distant metastatic sites. The absence of statistical significance for liver TBR_max_ when comparing primary tumours with lymph node metastases may be attributable to the more heterogeneous physiological liver uptake relative to the blood pool background.These findings are consistent with the biological concept that CAFs density and activation are most pronounced at the primary tumour site and may become more heterogeneous or attenuated during metastatic spread [2, 22]. On a lesion-based level, paired intra-patient comparisons of SUV_max_ and TBR_max_ confirmed significantly higher uptake in primary tumours compared to most avid metastatic sites. In contrast, the comparison of primary tumours and bone metastases did not reach statistical significance and was associated with small effect size, indicating largely comparable tracer uptake. This may reflect physiological stromal activity of the bone marrow or entity-specific difference in tumour-induced fibrosis [7, 22].

### Comparison with previous FAPI imaging studies and FDG-PET

Across entities with ≥ 5 analysed patients [^68^Ga]BED003 showed consistently high lesional uptake, with median SUV_max_ values between 10 and 20. This distribution is in close agreement with [^68^Ga]FAPI-02/04, where the highest average SUV_max_ (> 12) was reported for tumour types such as sarcoma, oesophageal, breast, and cholangiocarcinomas [7]. For the radiochemically comparable tracer [^68^Ga]FAPI-46, multiple-time-point clinical data (Naeimi et al. 2023) demonstrated stable tumour uptake and maintained comparable TBRs across a heterogeneous cohort of solid malignancies, supporting robust quantification. The overall uptake magnitude aligns well with the [^68^Ga]BED003 SUV_max_ and TBR_max_ medians observed in this study, e. g. for breast, oesophageal, pancreatic and ovarian cancer [23]. More recently, [^68^Ga]FAPI-2286 has been introduced with a high tumour uptake across a variety of solid malignancies (e. g. sarcoma, cholangiocarcinoma) with median SUV_max_ values that fall also within the range of [^68^Ga]BED003 [6]. With respect to [^18^F]fluorine labelled tracers, [^18^F]FAPI-74 has been evaluated for some different solid malignancies (e.g. breast, pancreatic cancer) with reported SUV_max_ values for primary tumours and metastases generally fall within a similar range to those observed in this study [9].

We found an intensive [⁶⁸Ga]BED003 uptake in cases of gynaecological cancers, including breast, ovarian, and cervical cancer. In recent investigations the histopathological evaluation of these malignancies revealed an intensive desmoplastic reaction in the context of gynaecological cancers, which provides a rationale for the intense fibroblast activation observed [7, 10, 24]. Furthermore, recent head-to-head comparison between [^68^Ga]FAPI-04 and [^18^F]FDG in gynaecological malignancies have demonstrated higher tracer uptake, and diagnostic accuracy for [^68^Ga]FAPI-04 [25].

Intensive binding of [^68^Ga]BED003 was observed in cases of hepatocellular, cholangiocellular and pancreatic cancers, as well as oesophageal and colorectal cancer. In line with the findings of preceding research, FAP-based imaging holds considerable promise for use in hepato-cholangiocellular-pancreatic and gastrointestinal malignancies in comparison to [^18^F]FDG-PET [7, 26]. It can be hypothesized that this is related to the low level of FAP binding in healthy upper abdominal and gastrointestinal organs compared to [^18^F]FDG, which results in a higher TBR [12]. Nevertheless, certain significant pitfalls have been identified as potential sources of variability. These include prolonged inflammatory reactions (e.g., liver cirrhosis, pancreatitis), the presence of benign lesions (e.g., liver haemangiomas) and pretreatments (e.g., surgery, radiation therapy) [15, 27, 28].

We investigated cases of medullary thyroid, salivary gland, and metastatic skin cancers (e.g., basal cell carcinoma) with known poor performance of [^18^F]FDG-PET [14, 26, 29]. These malignancies showed moderate to intense [^68^Ga]BED003 binding, consistent with the molecular characterization of FAP in the desmoplastic tumour stroma [30–32] and previous investigations of FAPI-PET [1, 7]. Moreover, preliminary research has shown that FAPI-PET is a reliable and effective method for imaging medullary thyroid cancer when compared to somatostatin receptor PET scans [33].

In addition, we reported malignancies with a low number of included patients (≤ 3), such as endometrial cancer or non–small cell lung cancer, showing moderate to intense tracer uptake. These results should be interpreted with caution due to selection and enrichment bias as well as the small patient numbers, despite the promising biodistribution of [^68^Ga]BED003. Physiological, non-tumour-related, and benign lesion–associated uptake of [⁶⁸Ga]BED003 was consistent with patterns previously reported for FAPI tracers [34].

### Implications for FAP-directed theranostics

Beyond diagnostic imaging, the observed uptake patterns have direct implications for FAP-targeted theranostics approaches. The broadly comparable uptake of [^68^ Ga]BED003 to established FAPI tracers suggests that [^68^Ga]BED003 PET imaging can reliably identify FAP-expressing tumours across multiple solid malignancies, thereby fulfilling a key prerequisite for patient selection in radioligand therapy. This is particularly relevant in the light of recent preclinical data on [^177^Lu]OncoFAP-23, which demonstrated prolonged tumour retention, favourable tumour-to-organ ratios, and potent antitumour efficacy in solid tumour bearing small animals [19]. Compared with monovalent FAPI radiotherapeutics, the multivalent architecture of OncoFAP-23 appears to overcome the rapid wash-out kinetics that may have limited therapeutic dose delivery in earlier approaches. [18, 19]. Importantly, the high and homogeneous tracer uptake observed with [^68^Ga]BED003 across the investigated lesions suggests that imaging-derived uptake metrics may serve as a non-invasive biomarker for tumour stromal target availability.

Early clinical experiences with other FAPI-based radioligand therapies, such as [^90^Y]FAPI-46, further support the clinical feasibility and acceptable safety profile of FAP-directed therapy in advanced and heavily pretreated solid malignancies [35]. While direct clinical data on [^177^Lu]OncoFAP-23 are lacking, the combination of robust preclinical efficacy, favourable biodistribution, and the imaging performance provides a strong rationale for clinical translation in the ongoing aforementioned clinical trial (NCT06640413). In this context, [^68^Ga]BED003 may not only serve as a sensitive imaging tool for detections of CAFs but also as a companion diagnostic to guide patient selection and treatment planning for OncoFAP-based radioligand therapy.

### Limitations

This investigation is limited by its retrospective approach. Lesions included in this analysis were classified as malignant primarily based on imaging findings and the intensity of fibroblast activation, which may have led to the inclusion of falsely positive lesions or the exclusion of falsely negative lesions. Selection and enrichment bias may have influenced the reported uptake values, since several patients were referred for FAPI imaging through an interdisciplinary tumour board. The absence of a direct comparison with well-established [^18^F]FDG PET in the same patients limits the interpretability of this study. However, the findings were compared with results from studies that have evaluated both tracers. Moreover, although the overall number of analysed patients is high, the number of patients for the different malignancies is rather low and underpowered to establish significant differences of tracer uptake amongst different tumour types.

## Conclusion

[^68^Ga]BED003-PET demonstrated consistently high uptake across a broad spectrum of solid malignancies, supporting its potential role as a tool for multi-cancer diagnostic imaging and patient selection for FAP-targeted radioligand therapy, e.g. with its multivalent derivative [^177^Lu]OncoFAP-23.

## Supplementary Information

Below is the link to the electronic supplementary material.

Supplementary file1 (DOCX 74 KB)

## Data Availability

The datasets generated during and/or analysed during the current study are available from the corresponding author on reasonable request.
