# Supplementary material for: FAP-targeted [68Ga]BED003-PET in different solid malignancies
Source: Eur J Nucl Med Mol Imaging. 2026 Mar 19;53(8):4977–87. doi: 10.1007/s00259-026-07842-1 (PMC13249670; doi:10.1007/s00259-026-07842-1)
Supplement: Supplementary file 1 — Supplementary file1 (DOCX 74 KB) [file 259_2026_7842_MOESM1_ESM.docx]

**SUPPLEMENTS**

**Figure 1S: Radiosynthesis of [^68^Ga]BED003 ([^68^Ga]OncoFAP-DOTAGA)**

**
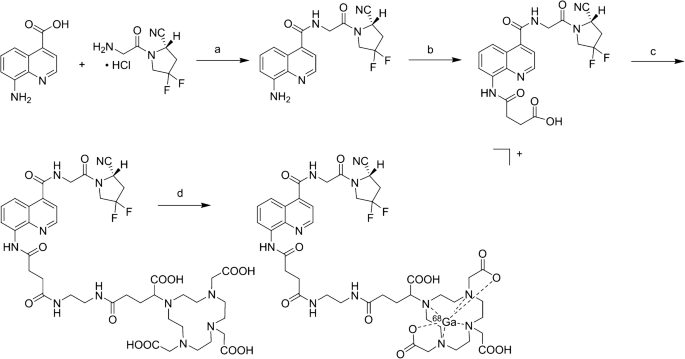
**

Radiosynthesis pathway illustration was adapted with permission from the publication “Backhaus, P et al. Translational Imaging of the Fibroblast Activation Protein (FAP) Using the New Ligand [^68^Ga]Ga-OncoFAP-DOTAGA. *Eur J Nucl Med Mol Imaging* 2022, *49*, 1822–1832”

**Table 1S:** All entities median SUV_max_ split by primary tumour, lymph node and organ metastases

| **Entity (n = lesions)** | | **Median SUV_max_** | **Range SUV_max_** |
| --- | --- | --- | --- |
| **Breast Cancer (n = 56)** | | **15.5** | **4.8–33.2** |
|  | Primary Tumour (n = 21) | 15.9 | 6.8–33.0 |
|  | Lymph Node Metastases (n = 16) | 17.0 | 4.8–33.2 |
|  | Distant Metastases (n = 19) | 13.6 | 6.5–23.1 |
| **Cholangiocellular Carcinoma (n = 25)** | | **15.6** | **6.8–35.9** |
|  | Primary Tumour (n = 9) | 21.8 | 14.5–35.9 |
|  | Lymph Node Metastases (n = 5) | 12.3 | 7.0–18.1 |
|  | Distant Metastases (n = 11) | 12.1 | 6.8–17.0 |
| **Cervical cancer (n = 9)** | | **17.8** | **5.0–31.4** |
|  | Primary Tumour (n = 4) | 23.7 | 17.9–31.4 |
|  | Lymph Node Metastases (n = 3) | 15.2 | 5.9–28.6 |
|  | Distant Metastases (n = 2) | 9.9 | 5.0–14.8 |
| **Colorectal Cancer (n = 20)** | | **13.3** | **5.2–23.6** |
|  | Primary Tumour (n = 1) | 20.0 | - / - |
|  | Lymph Node Metastases (n = 4) | 7.4 | 5.2–8.9 |
|  | Distant Metastases (n = 15) | 14.4 | 5.9–23.6 |
| **Cancer of unknown primary (n = 2)** | | **17.6** | **15.6–19.5** |
|  | Lymph Node Metastases (n = 2) | 17.6 | 15.6–19.5 |
| **Endometrial Cancer (n = 7)** | | **10.8** | **6.7–14.6** |
|  | Primary Tumour (n = 3) | 12.6 | 8.0–21.3 |
|  | Lymph Node Metastases (n = 1) | 6.8 | - / - |
|  | Distant Metastases (n = 3) | 10.2 | 6.7–13.1 |
| **Oesophageal Cancer (n = 25)** | | **16.7** | **4.4–28.8** |
|  | Primary Tumour (n = 16) | 17.9 | 8.5–28.8 |
|  | Lymph Node Metastases (n = 7) | 14.7 | 4.4–22.9 |
|  | Distant Metastases (n = 2) | 13.2 | 9.2–17.1 |
| **Hepatocellular Carcinoma (n = 24)** | | **13.6** | **5.0–28.2** |
|  | Primary Tumour (n = 15) | 15.9 | 5.1–28.2 |
|  | Lymph Node Metastasis (n = 4) | 11.9 | 7.1–20.2 |
|  | Distant Metastases (n = 5) | 8.1 | 5.0–15.7 |
| **Medullary Thyroid Cancer (n = 12)** | | **19.7** | **5.6–27.5** |
|  | Primary Tumour (n = 2) | 18.7 | 9.9–27.5 |
|  | Lymph Node Metastases (n = 4) | 11.9 | 5.6–16.6 |
|  | Distant Metastases (n = 6) | 9.3 | 6.7–15.7 |
| **Non-small-cell lung cancer (n = 2)** | | **19.7** | **18.1–21.3** |
|  | Primary Tumour (n = 1) | 18.1 | - / - |
|  | Lymph Node Metastasis (n = 1) | 21.3 | - / - |
| **Ovarian cancer (n = 17)** | | **15.3** | **6.5–22.9** |
|  | Primary Tumour (n = 5) | 15.9 | 8.5–22.9 |
|  | Lymph Node Metastases (n = 4) | 14.8 | 6.5–1.9 |
|  | Distant Metastases (n = 8) | 13.1 | 7.9–16.9 |
| **Pancreatic cancer (n = 54)** | | **13.4** | **4.5–29.7** |
|  | Primary Tumour (n = 26) | 16.9 | 8.8–29.7 |
|  | Lymph Node Metastases (n = 12) | 10.3 | 6.1–17.6 |
|  | Distant Metastases (n = 16) | 10.0 | 4.5–17.9 |
| **Parathyroid cancer (n = 3)** | | **12.9** | **9.4–19.5** |
|  | Primary Tumour (n = 1) | 19.5 | - / - |
|  | Distant Metastases (n = 2) | 9.5 | 9.4–9.7 |
| **Squamous cell carcinoma (n = 6)** | | **10.5** | **4.2–15.7** |
|  | Primary Tumour (n = 2) | 15.0 | 14.3–14.7 |
|  | Lymph Node Metastasis (n = 1) | 6.3 | - / - |
|  | Distant Metastases (n = 3) | 8.4 | 4.2–12.8 |
| **Salivary gland cancer (n = 12)** | | **14.6** | **8.9–22.6** |
|  | Primary Tumour (n = 3) | 14.8 | 10.6–18.9 |
|  | Lymph Node Metastases (n = 4) | 14.3 | 9.6–19.0 |
|  | Distant Metastases (n = 5) | 14.6 | 8.9–22.6 |
| **Sarcoma (n = 14)** | | **12.5** | **5.2–21.6** |
|  | Primary Tumour (n = 3) | 11.0 | 8.2–13.1 |
|  | Lymph Node Metastasis (n = 1) | 8.2 | - / - |
|  | Distant Metastases (n = 10) | 13.4 | 5.2–21.6 |
| **Skin cancer (n = 11)** | | **11.1** | **5.1–20.4** |
|  | Primary Tumour (n = 3) | 16.4 | 10.4–20.4 |
|  | Lymph Node Metastasis (n = 2) | 7.9 | 5.1–10.6 |
|  | Distant Metastases (n = 6) | 8.6 | 5.8–14.0 |
| **Small intestine cancer (n = 1)** | | **18.3** | **- / -** |
| **Urothelial cancer (n = 1)** | | **10.4** | **- / -** |

**Table 2S:** Lesion-based analysis with paired Wilcoxon signed-rank test for TBR_max_ values for comparison of primary tumour to lymph node and distant metastases

| **TBR_max_ Background** | **Comparison** | **Lesions** | **P value** | **Effect size (r)** |
| --- | --- | --- | --- | --- |
| Liver* | T > N | 46 | < 0.001 | 0.54 |
|  | T > M (lung) | 10 | 0.009 | 0.74 |
|  | T > M (liver) | 15 | 0.005 | 0.67 |
|  | T > M (bone) | 12 | 0.24 | 0.21 |
|  | T > M (other) | 16 | 0.022 | 0.5 |
| Blood pool | T > N | 48 | < 0.001 | 0.55 |
|  | T > M (lung) | 10 | 0.009 | 0.74 |
|  | T > M (liver) | 15 | 0.005 | 0.68 |
|  | T > M (bone) | 13 | 0.15 | 0.29 |
|  | T > M (other) | 16 | 0.02 | 0.5 |

*5 patients were excluded due to chronic liver disease

**Table 3S:** All entities median liver and blood pool TBR_max_ split by primary tumour, lymph node and organ metastases

| **Entity (n = lesions)** | | **Median Liver TBR_max_*** | **Range*** | **Median Blood pool**  **TBR_max_** | **Range** |
| --- | --- | --- | --- | --- | --- |
| **Breast Cancer (n = 56)** | | **24.7** | **7.9–55.4** | **11.1** | **3.7–33.4** |
|  | Primary Tumour (n = 21) | 23.2 | 9.6–29.6 | 11.3 | 5.4–30.7 |
|  | Lymph Node Metastases (n = 16) | 27.5 | 7.9–55.4 | 11.4 | 3.7–33.4 |
|  | Distant Metastases (n = 19) | 21.7 | 13.5–37.9 | 9.3 | 5–20.7 |
| **Cholangiocellular Carcinoma (n = 25)** | | **17.5** | **4.6–62.8** | **12.4** | **4.1–20.8** |
|  | Primary Tumour (n = 9) | 20.6 | 9.4–62.8 | 15.9 | 11.6–20.8 |
|  | Lymph Node Metastases (n = 4) | 27.5 | 4.6–32.9 | 8.8 | 4.1–18.9 |
|  | Distant Metastases (n = 11) | 15.5 | 5.3–47.2 | 9.6 | 4.6–17 |
| **Cervical cancer (n = 9)** | | **24.6** | **7.6–54.1** | **10.5** | **4.5–19.7** |
|  | Primary Tumour (n = 4) | 29.4 | 24.6–54.1 | 17.2 | 10.5–19.7 |
|  | Lymph Node Metastases (n = 3) | 15.8 | 8.9–43.3 | 6.6 | 5.3–17.3 |
|  | Distant Metastases (n = 2) | 15 | 7.6–22.4 | 6.7 | 4.5–9 |
| **Colorectal Cancer (n = 20)** | | **20.8** | **6.7–56.2** | **11.5** | **4.3–31.1** |
|  | Primary Tumour (n = 1) | 22 | -/- | 12 | -/- |
|  | Lymph Node Metastases (n = 4) | 9.5 | 9.3–23.3 | 5.9 | 4.3–12.9 |
|  | Distant Metastases (n = 15) | 20.9 | 6.7–56.2 | 12 | 5.3–31.1 |
| **Cancer of unknown primary (n = 2)** | | **31** | **27.9–34.2** | **15.1** | **10.8–19.3** |
|  | Lymph Node Metastases (n = 2) | 31 | 27.9–34.2 | 15.1 | 10.8–19.3 |
| **Endometrial Cancer (n = 7)** | | **17.2** | **8.8–34.4** | **8.6** | **4.4–17** |
|  | Primary Tumour (n = 3) | 20 | 10.5–34.4 | 8.8 | 5.2–17.1 |
|  | Lymph Node Metastases (n = 1) | 9 | -/- | 4.4 | -/- |
|  | Distant Metastases (n = 3) | 17.2 | 8.8–17.7 | 8.6 | 4.4–8.8 |
| **Oesophageal Cancer (n = 25)** | | **22.7** | **5.2–58.8** | **14.2** | **3.6–24.2** |
|  | Primary Tumour (n = 16) | 23.3 | 14.2–58.8 | 15.5 | 5.4–24.2 |
|  | Lymph Node Metastases (n = 7) | 22.7 | 5.2–33.5 | 11.9 | 3.6–23.4 |
|  | Distant Metastases (n = 2) | 23.9 | 18.8–29 | 12.4 | 11.6–13.2 |
| **Hepatocellular Carcinoma (n = 24)** | | **16.4** | **4.2–32.2** | **11.5** | **3.9–21** |
|  | Primary Tumour (n = 15) | 18.5 | 4.3–32.2 | 12.6 | 5–21 |
|  | Lymph Node Metastasis (n = 4) | 11.1 | 9.8–29.3 | 8.9 | 6.1–17.7 |
|  | Distant Metastases (n = 5) | 6.9 | 4.2–21.8 | 4.9 | 3.9–11.9 |
| **Medullary Thyroid Cancer (n = 12)** | | **16.1** | **3.6–35.7** | **8.8** | **5.8–22.2** |
|  | Primary Tumour (n = 2) | 26 | 16.2–35.7 | 15.6 | 8.9–22.2 |
|  | Lymph Node Metastases (n = 4) | 18.8 | 10–30.9 | 11.8 | 5.8–15.3 |
|  | Distant Metastases (n = 6) | 11.6 | 3.6–22.8 | 7.8 | 5.8–14.5 |
| **Non-small-cell lung cancer (n = 2)** | | **43.8** | **40.2–47.3** | **15.6** | **14.4–16.9** |
|  | Primary Tumour (n = 1) | 40.2 | -/- | 14.3 | -/- |
|  | Lymph Node Metastasis (n = 1) | 47.3 | -/- | 16.9 | -/- |
| **Ovarian cancer (n = 17)** | | **23.9** | **11–45.8** | **10.1** | **5.6–16** |
|  | Primary Tumour (n = 5) | 28.7 | 14.7–45.8 | 10.2 | 6.3–14.7 |
|  | Lymph Node Metastases (n = 4) | 28.3 | 11–39.6 | 10.9 | 5.6–16 |
|  | Distant Metastases (n = 8) | 17.5 | 12.5–33.5 | 9.6 | 6.3–13.9 |
| **Pancreatic cancer (n = 54)** | | **14.6** | **5.4–40.1** | **10.1** | **3–24.6** |
|  | Primary Tumour (n = 26) | 21.8 | 9.5–40.1 | 14 | 7.4–24.6 |
|  | Lymph Node Metastases (n = 12) | 10.7 | 6.2–28 | 6.5 | 4.4–15.9 |
|  | Distant Metastases (n = 16) | 12.5 | 5.4–30.9 | 7.8 | 3–19 |
| **Parathyroid cancer (n = 3)** | | **22.6** | **21.9–45.4** | **7.8** | **7.5–15.6** |
|  | Primary Tumour (n = 1) | 45.3 | -/- | 15.6 | -/- |
|  | Distant Metastases (n = 2) | 22.2 | 21.9–22.6 | 7.6 | 7.5–7.8 |
| **Squamous cell carcinoma (n = 6)** | | **18.4** | **7.1–33.3** | **10.5** | **4.4–17.2** |
|  | Primary Tumour (n = 2) | 25.5 | 17.6–33.3 | 14.1 | 11.1–17.2 |
|  | Lymph Node Metastasis (n = 1) | 7.1 | -/- | 4.4 | -/- |
|  | Distant Metastases (n = 3) | 19.1 | 9.8–29.8 | 9.9 | 5.1–15.4 |
| **Salivary gland cancer (n = 12)** | | **24.9** | **17.5–44.3** | **12.4** | **8.2–24.8** |
|  | Primary Tumour (n = 3) | 26.8 | 21.6–44 | 12.9 | 10.4–16.2 |
|  | Lymph Node Metastases (n = 4) | 29.4 | 21.2–43.2 | 13.9 | 8.2–17.6 |
|  | Distant Metastases (n = 5) | 23 | 17.5–44.3 | 11.1 | 9.7–24.8 |
| **Sarcoma (n = 14)** | | **19.2** | **5.4–30** | **10.6** | **3.8–17.5** |
|  | Primary Tumour (n = 3) | 23.4 | 8.5–26.2 | 10.8 | 5.5–14.4 |
|  | Lymph Node Metastasis (n = 1) | 16.4 | -/- | 5.4 | -/- |
|  | Distant Metastases (n = 10) | 19.2 | 5.4–30 | 10.6 | 3.5–17.9 |
| **Skin cancer (n = 11)** | | **14.7** | **6.7–29.8** | **8.4** | **3.8–17.5** |
|  | Primary Tumour (n = 3) | 28.3 | 15.5–29.8 | 16.1 | 9–17.5 |
|  | Lymph Node Metastasis (n = 2) | 10.7 | 6.7–14.7 | 6.1 | 3.8–8.4 |
|  | Distant Metastases (n = 6) | 9.5 | 7.6–19.4 | 5.4 | 4.3–11 |
| **Small intestine cancer (n = 1)** | | **20.5** | **-/-** | **14.6** | **-/-** |
| **Urothelial cancer (n = 1)** | | **16.5** | **-/-** | **7.4** | **-/-** |

*5 patients were excluded due to chronic liver disease
